# Comparative genomic analysis of ten *Elizabethkingia* *anophelis* isolated from clinical patients in China

**DOI:** 10.1128/spectrum.01780-24

**Published:** 2024-11-29

**Authors:** Fei Wu, Yan Wu, Miaomiao Zhang, Lijun Tian, Xue Li, Xi Luo, Yiquan Zhang, Renfei Lu

**Affiliations:** 1 Department of Clinical Laboratory, Nantong Third People’s Hospital, Affiliated Nantong Hospital 3 of Nantong University, Nantong, China; 2 Department of Critical Care Medicine, Nantong Third People’s Hospital, Affiliated Nantong Hospital 3 of Nantong University, Nantong, China; City of Hope Department of Pathology, Duarte, California, USA

**Keywords:** *Elizabethkingia anophelis*, next-generation sequencing, comparative genomics, capsule, iron

## Abstract

*Elizabethkingia anophelis* is an emerging pathogen that causes life-threatening infections in neonates and immunocompromised patients. In this study, we performed next-generation sequencing (NGS) to characterize 10 *E. anophelis* strains isolated from clinical patients in Nantong, China. Core, accessory, and unique genomes were composed of 2,891, 1,633, and 498 genes, respectively. Based on genetic screening for antimicrobial resistance genes (AMRs), all *E. anophelis* strains carried the same AMRs, including *bla*_B_, *bla*_CME_, and *bla*_GOB_. The virulence factors (VFs) in the 10 strains were classified into 13 functional categories, and the differences between strains were mainly in immune modulation and nutritional/metabolic factor. We further analyzed the genomic features of one of ten strains, NT06 strain. The capsule type of NT06 was X, which is rare among *E. anophelis* strains. Based on comparative analyses, we first found that NT06 carried the YclNOPQ-like operon, which is the complete transporter for petrobactin, to acquire iron. The genomic features are important for further investigations of epidemiology, resistance, virulence, and to identify appropriate treatments.

**IMPORTANCE:**

*Elizabethkingia anophelis* strains are opportunistic pathogens causing meningitis, bloodstream infections, and endophthalmitis in vulnerable populations. There is a lack of knowledge of the genetic diversity, presence of antimicrobial resistance genes (AMRs), and virulence factors (VFs) in *E. anophelis* isolated from clinical patient*s* in China. Based on next-generation sequencing (NGS) and comparative genomic analyses, we determined the genomic features, phylogeny, and diversity of *E. anophelis* strains isolated from patients and identified a large accessory genome, intrinsic AMRs, and variable VFs. Based on comparative analyses, we identified a key strain, NT06, that carried a unique capsule type of X and the siderophore-mediated iron acquisition system (*yclNOPQ*-like genes). These findings advance our understanding of the genomic plasticity, evolution, and pathogenicity determinants of *E. anophelis*.

## INTRODUCTION

*Elizabethkingia*, a Gram-negative, nonmotile, and aerobic bacillus, produces pale yellow colonies and can grow on MacConkey agar (1). *E. anophelis* was first identified in 2011, isolated from the midgut of the mosquito, *Anopheles gambiae* (2). *E. anophelis* is distributed in diverse natural environments, including water and raw milk (3, 4). Recently, an increasing number of studies have reported that *E. anophelis* was responsible for several outbreaks around the world, including America, France, Vietnam, Canada, and Taiwan (5–9). *E. anophelis* strains are associated with opportunistic infections in vulnerable populations, such as premature newborns and immunocompromised and critically ill patients, causing meningitis, bloodstream infections, and endophthalmitis (10–13).

*E. anophelis* carries intrinsic antimicrobial resistance genes (AMRs) encoded on the chromosome, including three β-lactamase genes (*bla*_B_, *bla*_CME_, and *bla*_GOB_) that confer resistance to the majority of β-lactam and aminoglycoside antibiotics (14). Multiple virulence factors (VFs) have also been identified in *E. anophelis*, including lipopolysaccharide (LPS), type IV pili, oxidative stress response genes, capsule, and siderophores (4, 15).

Iron is an essential cofactor for many enzymes that are involved in maintaining cellular homeostasis and function (16). Thus, the bioavailability of iron greatly influences bacterial metabolism, growth, and transcription. One of the most efficient strategies to sequester iron is to secrete iron-chelating siderophores (17). Aerobactin is a citrate–hydroxamate siderophore found in *E. anophelis* (15). It is a virulence factor that enables *E. anophelis* to sequester iron in iron-poor environments such as the urinary tract. The siderophore system of *E. anophelis* includes a synthesis gene cluster (*iucA*/*iucC*, *iucB,* and *iucD*) and specific siderophore receptors (TonB-dependent iron transports) involved in aerobactin siderophore biosynthesis and transport (15, 18). The objectives of this study were to use next-generation sequencing (NGS) data from 10 *E. anophelis* strains isolated from clinical patients in Nantong, China, to elucidate (i) genomic features, (ii) genetic diversity and evolutionary relationships among strains by constructing pan- and core genomes, (iii) the distributions of AMRs and VFs, (iv) comparative analyses of the capsule of the NT06 strain, and (v) the analyses of the PB uptake system (*yclNOPQ*-like) in the NT06 strain.

## RESULTS

### Clinical and genomic characterization of *E. anophelis* strains

Ten *E. anophelis* strains were isolated from clinical patients in Nantong Third People’s Hospital in 2023 (Table 1). Eight *E. anophelis* strains were isolated from male patients. All strains were isolated from elderly patients (average = 75.2 years; range = 64–89 years) with underlying diseases, such as hypertension, diabetes mellitus, and cholecystectomy. Most of the strains (9/10, 90%) were isolated from sputum from patients with serious pneumonia. All patients were in intensive care unit (ICU) and infectious disease department.

**TABLE 1:** Clinical information of the 10 *E. anophelis* strains

Characteristics	Value
Age (years)	
Range	64–89
Average	75.2
Sex (n)	
Male	8
Female	2
Ward	
Intensive care unit	5
Infectious disease department	5
Specimen (n)	
Sputum	10
Diagnosis (n)	
Pneumonia	9
Chronic renal insufficiency	1
Comorbidity (n)	
Hypertension	9
Diabetes mellitus	3
Cholecystectomy	3
Cerebral infarction	2
Heart disease	2
Chronic obstructive pulmonary disease	1
Respiratory failure	1
Pulmonary heart disease	1

The 10 *E. anophelis* genomes were sequenced using NGS, and the number of contigs ranged from 24 to 90, with an average of 69. The N50 ranged from 136,198 bp to 562,042 bp, with an average of 247,351 bp. The number of predicted CDSs ranged from 3,590 to 3,662, with an average of 3,637 genomes. We calculated the genome completeness (average = 99.99%; range = 99.7 – 100%) and genome contamination (average = 0.06%; range = 0 – 0.28%), which were within the genome quality standards recommended by CheckM2 (Table 2). Whole-genome pairwise sequence comparisons revealed an average nucleotide identity (ANI) ranging from 97.68% to 98.37% for the most distant and the closest strains, respectively, compared to the *E. anophelis* NUHP1 strain (CP007547.1) (Fig. S1).

**TABLE 2:** Genome characteristics of the 10 *E. anophelis* strains

Strain	Genome size (bp)	No. of contigs	N50	L50	G + C content (%)	CDS	tRNA	rRNA	Completeness/contamination (%)	Types of ICE*Ea*
NT01	3,983,032	81	137,911	9	35.57	3,647	45	3	100/0	ICE*Ea*II
NT02	3,982,680	81	136,198	10	35.58	3,652	45	3	100/0	ICE*Ea*II
NT03	3,983,031	81	137,911	9	35.57	3,652	45	3	100/0	ICE*Ea*II
NT04	3,983,010	82	136,198	10	35.57	3,648	45	3	100/0	ICE*Ea*II
NT05	3,957,455	80	365,975	4	35.55	3,662	47	3	100/0.28	–
NT06	3,939,226	24	562,042	4	35.61	3,590	46	3	100/0	ICE*Ea*II
NT07	3,946,750	45	247,583	5	35.56	3,603	46	3	99.97/0.05	ICE*Ea*I
NT08	3,955,394	90	365,973	5	35.55	3,658	46	3	100/0.24	–
NT09	3,946,774	44	247,524	5	35.56	3,608	46	3	99.97/0.05	ICE*Ea*I
NT10	3,982,613	81	136,198	10	35.58	3,653	45	3	100/0	ICE*Ea*II

The integrative and conjugative elements (ICEs) of *E. anophelis* were also analyzed, named as ICE*Ea*. Type I ICE*Ea* was identified in NT07 and NT09 strains. Type II ICE*Ea* was recognized in NT01, NT02, NT03, NT04, NT06, and NT10 strains. The NT06, NT07, and NT09 strains lacked the *traO* and *traQ* genes. ICEs could not be determined in NT05 and NT08 strains because of the interrupted sequence (Table 2).

### Pan-genome analysis

**Fig 1:**
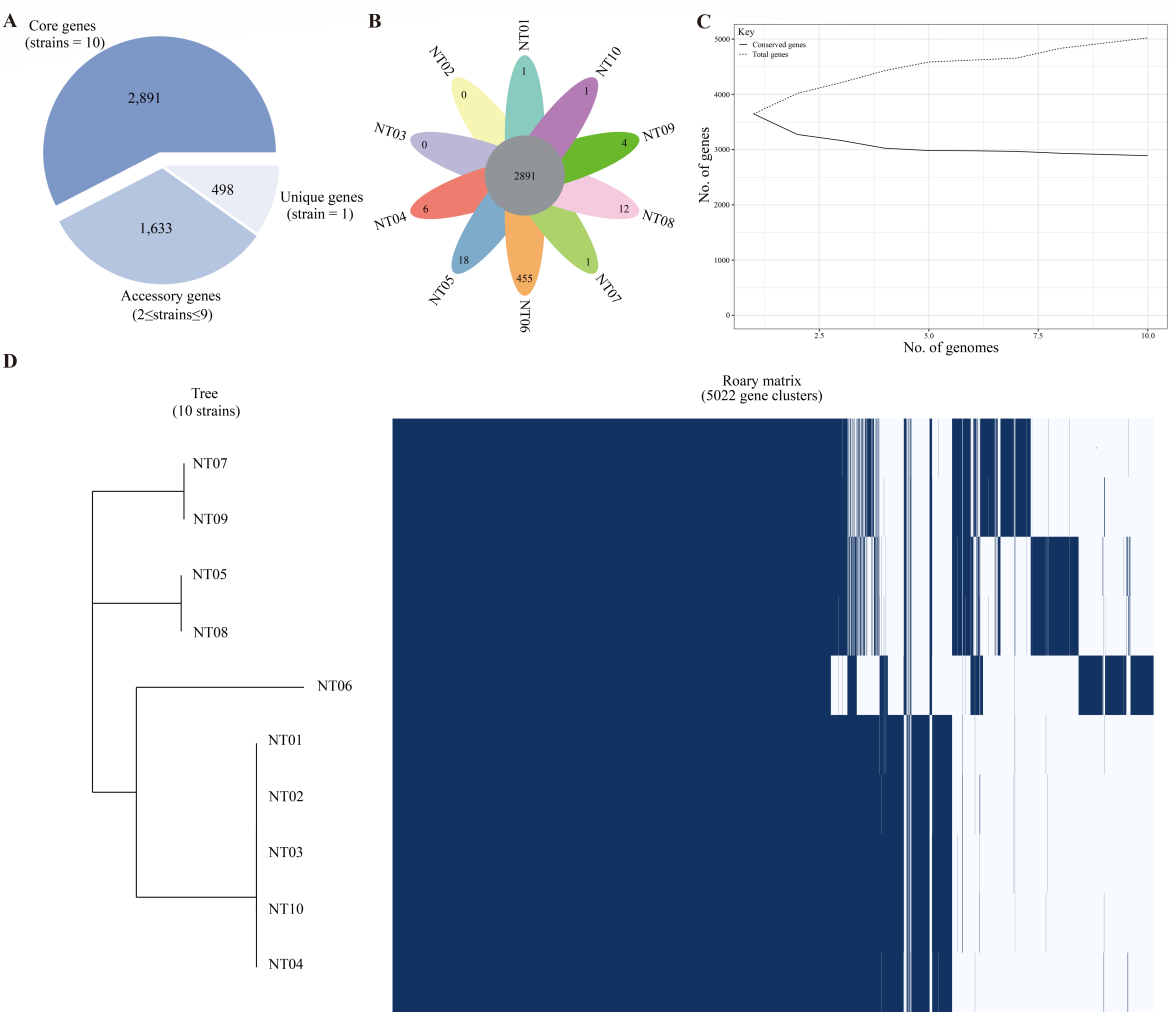
Pan-genome analysis of 10 *E. anophelis* strains conducted using Roary. (**A**) Pie chart of the proportion of core, accessory, and unique genes in the pan-genome. (**B**) Flower plot shows the number of core genes and unique genes in each *E. anophelis* strain. (**C**) The size of the core genome and pan-genome with increasing numbers of *E. anophelis* genomes. (**D**) The genetic presence–absence profile and cluster result of *E. anophelis* strains.

The pan-genome of the 10 *E. anophelis* strains had 5,022 genes. The core genome (genes present in all 10 *E. anophelis* strains) consisted of 2,891 genes. The accessory genome (shared by ≥2 strains, but not all) consisted of 1,633 genes, and the unique genome (genes in only one strain) was composed of 498 genes (Fig. 1A). The NT06 strain contained the most unique genes (455, 91.73%) compared to the other nine strains (Fig. 1B). The pan-genome fitted cumulative curve revealed that with the addition of more genomes, the number of core genes remained relatively stable, while the number of total genes continued to increase, thus indicating that the pan-genome is still open (Fig. 1C). The Roary matrix based on the presence and absence of the genes among the 10 *E. anophelis* genomes was also generated (Fig. 1D). NT07 and NT09 belonged to the same clade, while NT05 and NT08 were clustered into the same clade. The largest clade consisted of six *E. anophelis* strains, namely, NT06, NT01, NT02, NT03, NT10, and NT04, while NT06 belonged to a subclade of other five strains.

Functional annotation of the genes in the pan-genome was performed using the COG and KEGG databases and revealed a distribution of functional categories among three pan-genome sets (Fig. S2). The functions of amino acid metabolism, carbohydrate metabolism, and the metabolism of cofactors and vitamins were enhanced in the core genome, whereas the functions of glycan biosynthesis and metabolism, carbohydrate metabolism, signal transduction, and cellular community were enhanced among the KEGG functional pathways in the unique genome (Fig. S2A). The COG functional categories enriched in the core genome included amino acid transport and metabolism, transcription, and cell wall/membrane/envelope biogenesis. The COG categories enriched in the unique genome included cell wall/membrane/envelope biogenesis, transcription, and inorganic ion transport and metabolism (Fig. S2B). The unique genes in the NT06 strain were mainly involved in glycan biosynthesis and metabolism, cell wall/membrane/envelope biogenesis, transcription, and inorganic ion transport and metabolism (Tables S1 and S2).

### Antimicrobial resistance genes and virulence factors

**Fig 2:**
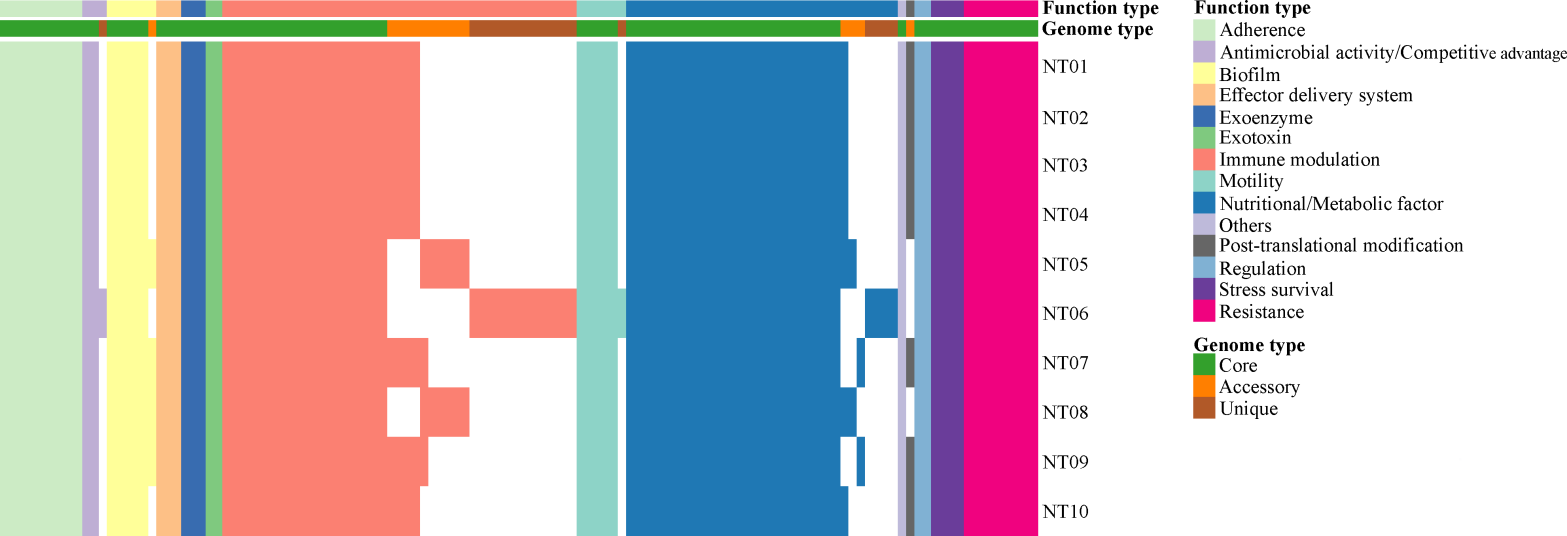
Presence/absence pattern of AMRs and VFs in each *E. anophelis* genome.

Nine AMRs were predicted among the 10 strains, including β-lactam resistance genes (*bla*_B,_ *bla*_CEM,_ and *bla*_GOB_), vancomycin resistance genes (*vanT* and *vanW*), quaternary ammonium compound resistance genes (*qacG*), and resistance nodulation cell division (RND) antibiotic efflux pump (three copies of *adeF*) (Fig. 2). The antimicrobial susceptibility between all 10 *E. anophelis* strains is shown in Table 3. All *E. anophelis* strains were resistant to tobramycin, imipenem, meropenem, aztreonam, ticarcillin–clavulanate, ceftazidime, cefepime, and polymyxin B. The resistance rates to trimethoprim–sulfamethoxazole, cefoperazone–sulbactam, and tigecycline were very low. All strains were susceptible to minocycline and exhibited intermediate resistance to vancomycin.

**TABLE 3:** Antimicrobial susceptibility of the 10 *E. anophelis* strains^*a*^

Antibiotics	NT01	NT02	NT03	NT04	NT05	NT06	NT07	NT08	NT09	NT10
Amikacin	R	R	R	R	R	R	R	R	S	R
Tobramycin	R	R	R	R	R	R	R	R	R	R
Imipenem	R	R	R	R	R	R	R	R	R	R
Meropenem	R	R	R	R	R	R	R	R	R	R
Aztreonam	R	R	R	R	R	R	R	R	R	R
Piperacillin–tazobactam	I	S	R	R	R	S	R	S	S	I
Ticarcillin–clavulanate	R	R	R	R	R	R	R	R	R	R
Trimethoprim–sulfamethoxazole	S	S	S	S	R	S	R	S	R	S
Ciprofloxacin	R	R	R	R	S	I	R	S	S	R
Levofloxacin	R	R	R	R	S	S	R	S	S	R
Ceftazidime	R	R	R	R	R	R	R	R	R	R
Cefepime	R	R	R	R	I	R	R	R	R	R
Cefoperazone–sulbactam	S	S	S	S	S	S	R	S	S	S
Minocycline	S	S	S	S	S	S	S	S	S	S
Tigecycline	S	S	S	S	I	S	R	S	S	S
Polymyxin B	R	R	R	R	R	R	R	R	R	R
Vancomycin	I	I	I	I	I	I	I	I	I	I

^*a*^ S, susceptible; R, resistant; I, intermediate.

A total of 117 VFs were predicted among the 10 *E. anophelis* strains, and the most of VFs (83, 70.94%) were encoded by core genes. These VFs were mainly associated with nutritional/metabolic factor (heme biosynthesis), immune modulation (capsule, lipooligosaccharide, and LPS), and adherence (type IV pili) (Fig. 2). The ferric aerobactin receptor gene (*iutA*) was detected in all strains. The most differences among the strains were mainly in immune modulation (lipooligosaccharide) and nutritional/metabolic factor (heme biosynthesis). The unique genes in NT06 were involved in immune modulation (capsule and LPS) and nutritional/metabolic factor (ferric siderophore) (Fig. 2).

### Comparative analysis of the capsule of the NT06 strain

Since the most unique genes in the NT06 strain (139, 30.55%) were encoded by contig4, that contig was used as the reference to compare all contig sequences of the other nine strains (Fig. S3). Compared to the other nine strains, several unique genomic regions (GRs) ranging from 9 kbp to 30 kbp were observed in NT06 (Fig. S3). Such GRs included genes for O-antigen polysaccharide polymerase in GR2, genes for AAA family ATPases in GR3, genes for conjugal transfer and the siderophore-interacting system in GR5, and phosphatase and glycosyltransferase in GR6 (Table S3).

An identical Wzy-dependent capsular polysaccharide synthesis (*cps*) cluster was found in GR2. By BLASTn with *E. anophelis* sequences in the NCBI database, the *cps* cluster type of the NT06 strain was X, according to the study by Amandine Perrin (19). Three other *cps* cluster type X of *E. anophelis* strains, including *E. anophelis* 502 isolated from a clinical patient in the United Kingdom and *E. anophelis* SKLX024812 and *E. anophelis* SKLX033378 isolated from clinical patients in China, were identified in the NCBI database. We further analyzed the *cps* cluster of the other nine *E. anophelis* strains. The NT01, NT02, NT03, NT04, and NT10 strains contained *cps* gene cluster type XIb, NT07 and NT09 contained *cps* gene cluster type I, and NT05 and NT08 contained *cps* gene cluster type XII.

**Fig 3:**
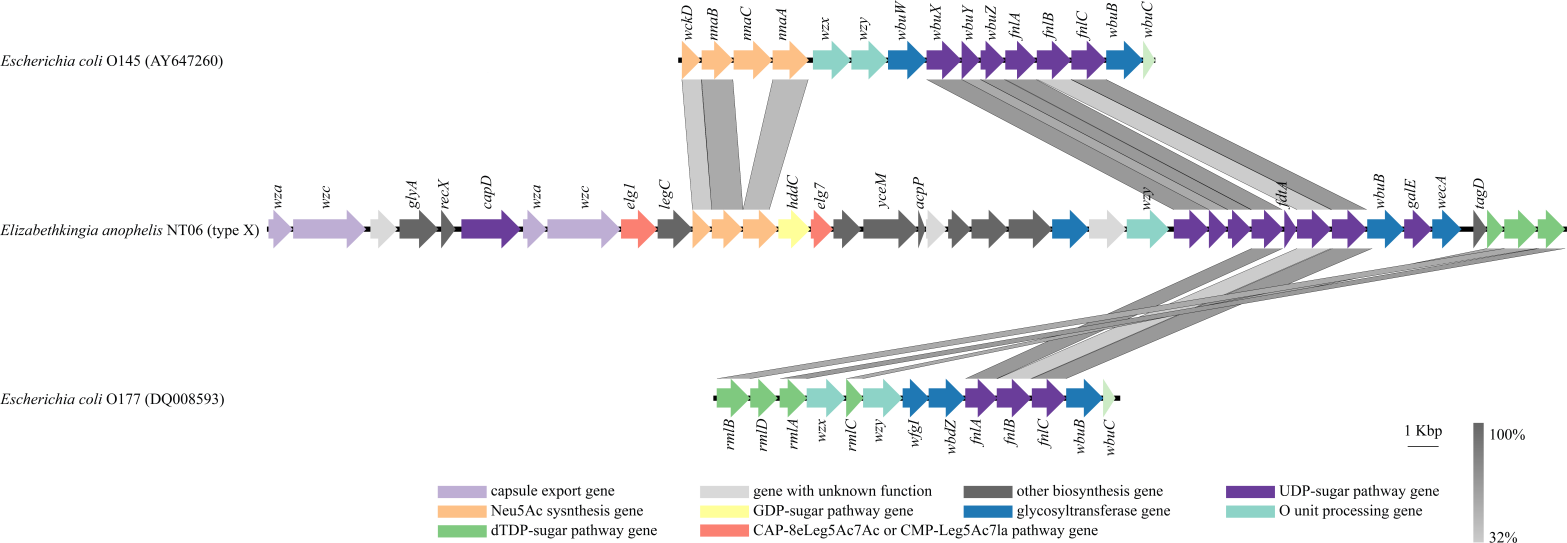
Comparative analysis of the *cps* cluster of the NT06 strain, *E. coli* O145, and *E. coli* O177. The location and polarity of CDSs are shown with arrows. The extent of homologous regions is indicated in the dark gray shading, as compared with BLASTp.

The *cps* cluster of the NT06 strain was about 42 kbp, encoding 39 genes. The O-antigen clusters have been studied well in *Escherichia coli*. The comparative sequence analysis with *E. coli* O-antigen sequences showed that the *cps* cluster of NT06 was similar to that of *E. coli* O145 containing Neu5Ac synthesis genes (*wckD* and *nnaAB*) and UDP–sugar pathway genes (*wbuXYZ* and *fnlABC*), with identities ranging from 32.09% to 70.29 by BLASTp. The cps cluster of NT06 was similar to that of *E. coli* O177 containing dTDP–sugar pathway genes (*rmlABD*) and UDP–sugar pathway genes (*flnABC*) with identities ranging from 32.71% to 69.54 by BLASTp (Fig. 3). Several capsule genes (*wza* and *wzc*) and other biosynthesis genes (*glyA*, *recX,* and *legC*) upstream of *nna* genes in the NT06 strain. The genes between *nna* and *wbu* genes were mainly involved in other biosynthesis functions, and the genes (*wbuB* and *wecA*) involved in glycosyltransferase function were between the *fnl* and *rml* genes in the NT06 strain.

### Comparative analysis of siderophore cluster genes in the NT06 strain

The GR5 was the largest GR in contig4 of the NT06 strain, containing mobilization relaxosome genes (*mobABC*) and siderophore cluster-like genes (*yclNOPQ*-like). This is the first study reporting that the siderophore cluster-like proteins, including the transporter YclO, ATPase YclP, permease YclN, and substrate-binding protein YclQ, found in an *E. anophelis* strain. The *ycl*-like cluster genes in NT06 were very similar to Ycl proteins in *Bacillus subtilis*, with the coverage ranging from 88.29% to 99.21% and identity ranging from 39% to 51% by BLASTx (Table S4). Two other *E. anophelis* strains SZ27 and SZ28, which encoded *yclNOPQ*-like cluster genes, were also found in the NCBI database by BLASTn. These two *E. anophelis* strains were both isolated from throat swab sample kits in Shenzhen, China.

**Fig 4:**
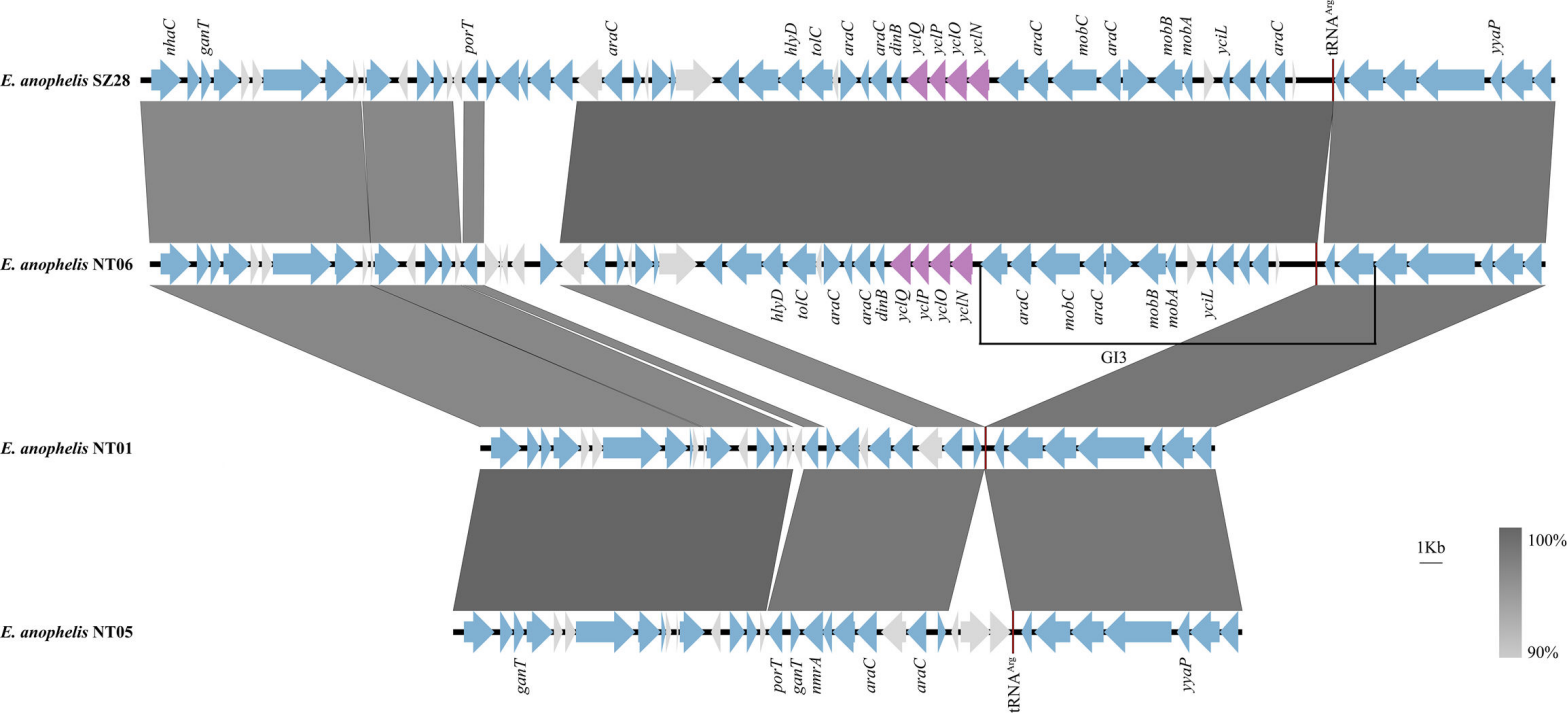
Comparative genomic analysis of the genetic context of *yclNOPQ*-like genes in the NT06 strain with the sequences of three other *E. anophelis* strains. The location and polarity of CDSs are shown with arrows. The extent of homologous regions is indicated in the dark gray shading.

The sequences of SZ28, NT01, and NT05 strains were used as representative sequences to compare against the NT06 sequence (Fig. 4). The NT06 sequence containing the *yclNOPQ*-like cluster genes was almost the same, as SZ28, except for several genes downstream of *porT*. Both SZ28 and NT06 strains contained mobilization genes (*mobA*, *mobB,* and *mobC*). The mobilization relaxosome (*mobABC*) cluster genes that are downstream of *yclNOPQ*-like were in a genetic island (GI), GI3. Compared to the NT01 strain, the sequences containing the *yclNOPQ*-like cluster genes and *mobABC* were integrated upstream of the tRNA^Arg^ locus. The other genes with unknown functions were also integrated at the tRNA^Arg^ locus in the NT05 strain, compared to the NT01 strain.

**Fig 5:**
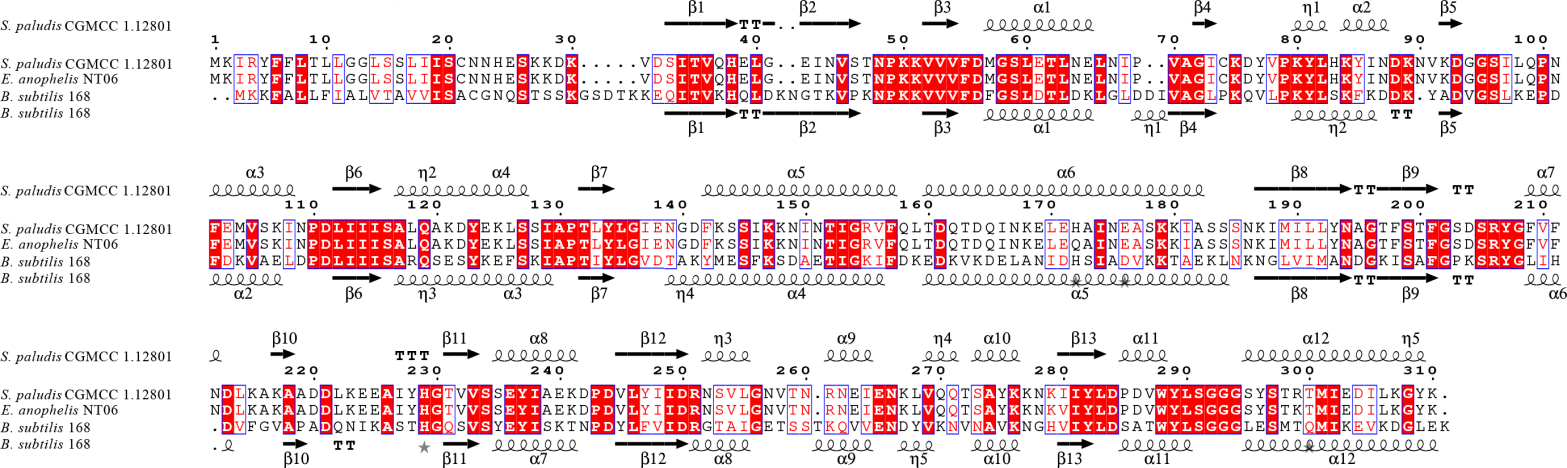
The secondary structure of YclQ-like protein and its homologs in *S. paludis* CGMCC 1.12801 and *B. subtilis* 168. Multiple sequence alignments were performed using the ClustalW program within MEGA 11. The protein secondary structure of YclQ in *S. paludis* CGMCC 1.12801 and *B. subtilis* 168 are labeled on the top and bottom, respectively. Structural elements, such as α-helices, turns (**T**), and β-strands (arrows), are indicated. Residues strictly conserved have a colored background and are indicated by boldface letters; residues conserved between groups are boxed.

The modeling of the YclQ-like protein was based on the published iron complex transport system substrate-binding protein (template number A0A4R7CYS8.1.A) and petrobactin-binding protein YclQ (template number 3gfv.1.A), with sequence identities of 99.03% and 39.64% by BLASTp and global model quality estimation (GMQE) scores of 0.9 and 0.72, respectively. The secondary structures of the YclQ-like protein and its homologs in *Sphingobacterium paludis* CGMCC 1.12801 and *Bacillus subtilis* 168 were aligned to each other (Fig. 5). The alignment data showed that the secondary structures of YclQ-like proteins were almost the same.

The YclQ-like protein was similar to proteins in other strains, such as *Sphingobacterium paludism*, *Elizabethkingia meningoseptica*, and *Sphingobacterium* sp with 100% coverage and identities ranging from 98.39% to 100%. One hundred and thirty-nine YclQ-like protein sequences encoded by 137 strains (including the NT06 strain) were used for further analysis by BLASTx, with coverage ≥90% and identity >30% (Table S5). The most genera of 137 strains were *Acinetobacter* (67, 48.91%) and *Bacillus* (24, 17.52%). Meanwhile, most strains (59, 43.07%) were isolated from the environment, and 35 strains were isolated from humans.

**Fig 6:**
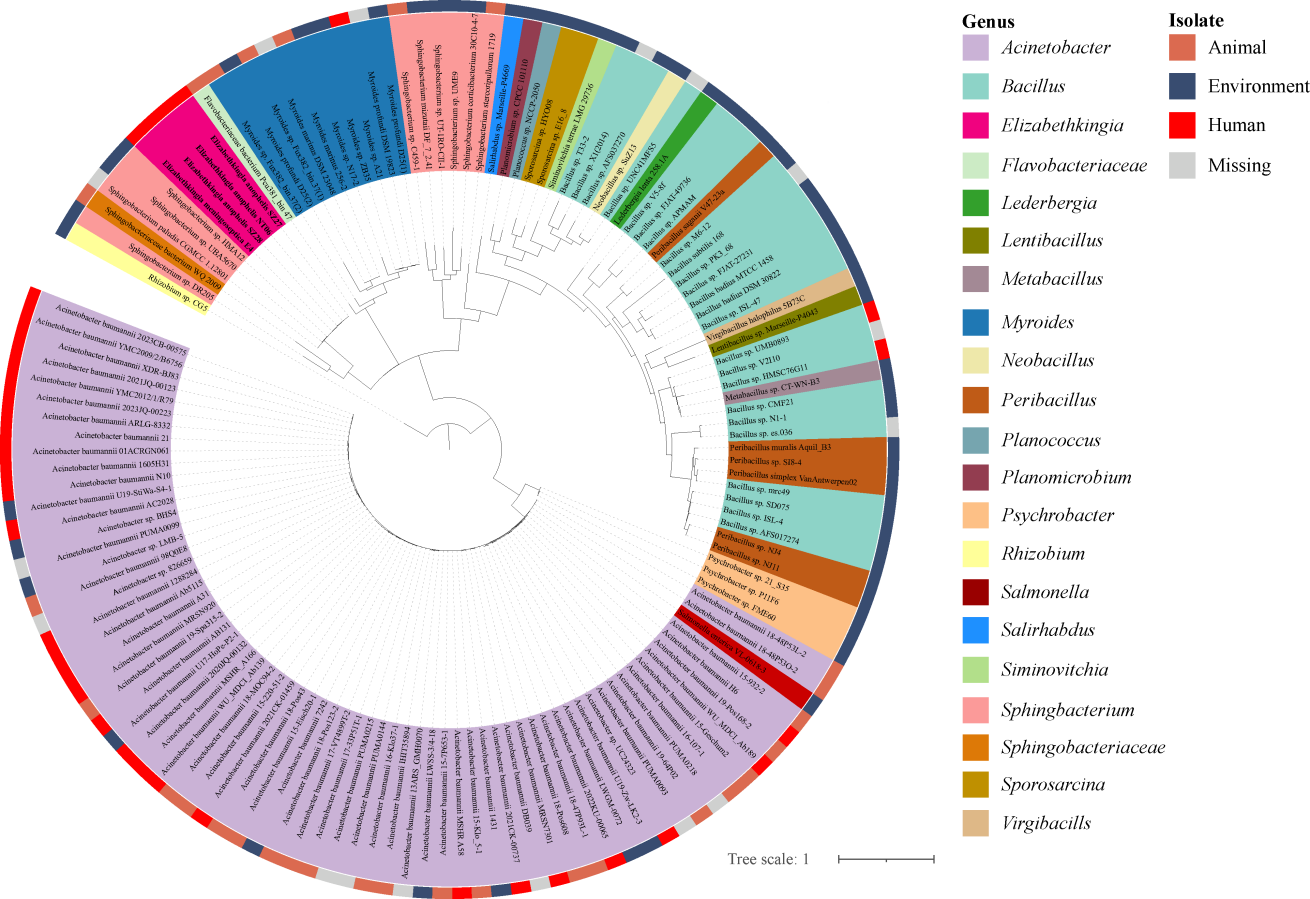
Maximum likelihood tree based on 139 YclQ protein sequences from 137 strains. The protein sequences were aligned using MAFFT, and the alignments were trimmed using trimAl. The tree was made using IQ-TREE, and *R. sp*. CG5 was used as the outgroup. The different colors represent different genera and isolations, as defined in the legend.

A phylogenetic tree was constructed using the 139 YclQ-like proteins in 137 strains (Fig. 6). The same genus of bacteria was almost entirely sorted in one clade, such as that for *Acinetobacter*, *Bacillus*, and *Myroides*. The phylogenetic tree of the YclQ-like protein showed that three *E. anophelis* strains (NT06, SZ27, and SZ28), *Elizabethkingia meningoseptica* E4, two *Sphingobacterium* sp. strains (HMA12 and UBA5670), and *Sphingobacterium paludis* CGMCC 1.12801 were clustered in one clade. All genus *Elizabethkingia* strains were isolated from humans in China, while two *Sphingobacterium* sp. strains were isolated from the environment and *Sphingobacterium paludis* CGMCC 1.12801 were missing.

One hundred and twenty-two YclNOPQ-like proteins in 122 strains were used to construct a concatenated protein tree. The concatenated protein tree was almost the same as the one based on the YclQ protein, with the genus *Elizabethkingia*, two *Sphingobacterium* sp. strains (HMA12 and UBA5670), and *Sphingobacterium paludis* CGMCC 1.12801 clustered in one clade (Fig. S4).

## DISCUSSION

In this study, we identified 10 *E. anophelis* strains isolated from clinical patients in Nantong, China. Most of *E. anophelis* strains were isolated from the elderly patient (ranging from 64 to 89), and half of the patients were in the ICU department. These results were consistent with the results of a study in Taiwan (9). Most patients with *E. anophelis* infections were reported to suffer comorbidities, such as solid-organ tumor, diabetes mellitus, and hematologic malignancy (9). All patients in our study also had at least one underlying disease, such as hypertension, diabetes mellitus, cholecystectomy, and cerebral infarction. The mortality rate of patients with *E. anophelis* infections ranged from 23.5% to 34.2% (20). In our study, one patient infected with the NT08 strain, was dead, and the low case-fatality rate may result from the limitation of the infection case.

The development of next-generation sequencing (NGS) technologies has made the pan-genome a new tool for analyzing pathogenic bacteria (21). We compared the genome sizes, the G + C content, and the number of genes reported for the *E. anophelis* strains deposited in the GenBank database, finding that the values obtained for our sequenced strains were very close to those reported previously (9, 22). Though the ANI value of NT06 is low compared with that of other *E. anophelis* strains, the ANI value > 95% indicated the same species (23). The phylogenetic tree of the 16S rRNA gene shows that all *E. anophelis* strains were clustered in the same group (Fig. S5). Those data suggest that our assemblies and annotations had generated fragments, but were close to complete genomes.

Integrative and conjugative elements (ICEs) are capable of horizontal transfer between bacteria via conjugation (24). Three types of ICE*Ea* were found in the 10 *E. anophelis* strains. ICE*Ea* in NT06, NT07, and NT09 strains lacked the *traO* and *traQ* genes, confirmed by the result of the CR. TraO is a putative surface adhesin, which enhances the cell-to-cell contact between donor and recipient cells prior to conjugative transfer (25). TraQ may be a repressor protein that controls the expression of transfer genes (26). Those two genes may have been lost after conjugative transfer.

Plasmid sequences were not detected, and nine resistance genes were detected as core genes on the chromosome of all 10 strains. β-lactamase genes (*bla*_B_, *bla*_CME_, and *bla*_GOB_) were intrinsic resistance genes in *E. anophelis*, as also reported in previous studies (27–29). There were no inserted sequences or transposases identified around AMRs. We hypothesize that the four AMRs (*vanT*, *vanW*, *qacG*, and *adeF*) thought to be related to resistance to vancomycin, fluoroquinolone, and quaternary ammonium compounds were also intrinsic genes on the chromosome (30–32). Most of the 10 strains were resistant to β-lactams (meropenem, aztreonam, and imipenem), aminoglycosides (amikacin), and cephalosporins (ceftazidime and cefepime). All 10 strains exhibited intermediate resistance to vancomycin. The previous study reported that the intermediation and resistance rate of vancomycin was about 74.11% and 25.38%, respectively (33). The presence of multiple resistance genes contributes to the multidrug resistance of *E. anophelis* and increases the difficulty of clinical treatment.

Seventeen different *cps* cluster types were defined based on their gene composition pattern (19). The region of the *cps* locus that encoded secretory proteins such as Wza and Wzc was highly conserved in *Elizabethkingia*, whereas the proteins involved in generating the specific polysaccharidic composition of the capsule were encoded in a highly variable region (34). The presence of variable *cps* clusters suggests an important determinant of the pathogenicity mechanisms and virulence heterogeneity among *E. anophelis* strains (34). The *cps* gene cluster type X was found in NT06 and three other *E. anophelis* strains in the NCBI database. All of the type X *cps* gene clusters of *E. anophelis* strains were isolated from clinical patients, and three of which were isolated in China. The capsular polysaccharide may play a possible pathogenic role in the outbreak of the strain (19). Thus, few *cps* gene cluster type X were observed in *E. anophelis*, and this cluster type must still be monitored in clinical patients in China.

The mobilization genes (*mobABC*) and tRNA^Arg^ were found in GI3 in the NT06 strain. The nickases MobA and auxiliary MobB and MobC were necessary for the initiation and termination stages of conjugal transfer (35). The tRNA genes are often the targets of site-specific recombinases that transfer genomic islands to the chromosomes of recipient bacteria (36). The sequence containing *yclNOPQ* and *mobABC* may be inserted at the tRNA^Arg^ locus. The CDSs between *araC* and tRNA^Arg^ were highly homologous with those of *Flavobacterium dauae* TCH3-2.

Many iron uptake-related genes were detected in *E. anophelis* strains, such as the siderophore receptor (*tonB*), yersiniabactin-like siderophore (*iucA*_*iucC*/*iucB*/*iucD*), and hemin utilization genes (15, 18). All such iron uptake-related genes were found in 10 *E. anophelis* strains. The siderophore cluster-like genes (*yclNOPQ*) were found in *E. anophelis* NT06. Petrobactin is a bis-catechol siderophore and a crucial virulence factor in *Bacillus anthracis* that enables the bacterium to establish infection in mice and supports growth in macrophages (37). The study reported that *Bacillus subtilis* can use petrobactin (PB) to acquire iron via the *yclNOPQ* operon (38). Isogenic disruption mutants of the *yclNOPQ* transporter, including permease YclN, ATPase YclP, and the substrate-binding protein YclQ, are unable to use either PB or the photoproduct of FePB for iron delivery and growth (39).

The *yclNOPQ*-like genes were located upstream of GI3 in the NT06 strain, and this is the first study reporting that *yclNOPQ*-like genes were found in *E. anophelis*. The *yclNOPQ*-like cluster genes and CDSs in GI3 may be integrated into the tRNA^Arg^ site together. The resulting model of the YclQ-like protein was based on the iron complex transport system substrate-binding protein (template number A0A4R7CYS8.1.A) and petrobactin-binding protein, YclQ (template number 3gfv.1.A), with the global model GMQE score of 0.9 and 0.72, respectively. Those data were indicative of good reliability and accuracy (https://swissmodel.expasy.org/docs/help).

By comparative analysis, we found that YclNOPQ-like proteins were not only observed in *Elizabethkingia* but also in the other genera, such as *Sphingobacterium*, *Myroides,* and *Acinetobacter*. Two other *E. anophelis* strains (SZ27 and SZ28) isolated from clinical patients in Shenzhen harbored *yclNOPQ*-like genes by BLASTn in the NCBI database. The YclNOPQ-like proteins may comprise the virulence-associated PB-mediated iron acquisition systems in *E. anophelis* and could be a target for novel antibacterial approaches. Much more work is required to be done to study well the function of YclNOPQ-like proteins in *E. anophelis* strains.

In summary, by pan-genome and comparative analyses, we studied the distribution of AMRs and VFs among 10 *E. anophelis* strains isolated from patients in Nantong, China. We identified a unique strain, NT06, that contains the rare *cps* cluster X and PB uptake system (YclNOPQ-like proteins). This is the first study reporting the specific PB uptake system in an *E. anophelis* strain. This PB uptake system may play important roles in survival and virulence in *E. anophelis* strains.

## MATERIALS AND METHODS

### Bacterial isolates and genomic DNA extraction

Ten clinical *E. anophelis* strains collected in Nantong, China, in 2023 were included in this study. The strains were identified using the bioMérieux VITEK 2 compact instrument (bioMérieux, Marcy-l’Étoile, France) and ANI analysis. The antimicrobial susceptibility was determined using the Vitek 2 system and susceptibility testing cards (bioMérieux, Lyon, France). The breakpoints for antimicrobial susceptibility were interpreted using the criteria for other non-*Enterobacterales* according to CLSI M100-S30. The breakpoint for vancomycin was adapted from the CLSI criteria for *Enterococcus* species.

The genomic DNA of the 10 *E. anophelis* strains was extracted using the TIANamp Bacteria DNA Kit (Tiangen Biotech Company Ltd., Beijing, China), according to the manufacturer’s protocol. The primers for *traO* and *traQ* genes were designed using Primer Premier6.0.

### Whole-genome sequencing, genome assembly, and annotation

The extracted genomic DNA was sequenced using the Illumina MiSeq system (Sanigen, South Korea). After genome sequencing, the qualified raw sequence reads were used for genome assembly using Unicycler v0.5.0 (40). QUAST v5.2.0 was used to assess the quality of each resulting assembly (41), and CheckM2 v1.0.1 was used to evaluate genome contamination/completeness (42). Annotation of the genome was performed using Prokka v1.14.6 with default settings (43). The coding sequences (CDSs) of interest were annotated manually using the UniProtKB/SWISS-Prot database (44). The rRNA and tRNA sequences were annotated using RNAmmer and tRNAscan-SE, respectively (45, 46). The Kyoto Encyclopedia of Genes and Genomes (KEGG) and Cluster of Orthologous Groups (COG) were analyzed using KofamScan v1.3.0 and eggNOG-mapper (http://eggnog-mapper.embl.de/), respectively (47, 48). AMRs were identified using the Resistance Gene Identifier (RGI) v6.0.3 (https://github.com/arpcard/rgi) and ABRicate v.1.0.0 (https://github.com/tseemann/abricate) against the comprehensive antibiotic resistance database (CARD) (49). The sequences of 10 strains were annotated and compared using the Basic Local Alignment Search Tool (BLAST) tool, BLAST +2.9.0 (50). Putative VFs were identified using the virulence factors database (VFDB_setB) by BLASTx (51). To identify ICEs, the annotations of 10 genomes were searched for clusters of genes coding for an integrase, relaxase, coupling protein (T4CP), and transfer (Tra) proteins, including a VirB4 ATPase (TraG) in the conjugation module (24). Genomic islands (50) were identified using the online tool, IslandViewer 4 (52).

### Genomic analyses

The whole-genome ANI between pairwise *E. anophelis* strains was calculated by FastANI v1.33 using the *E. anophelis* NUHP1 strain (CP007547.1) as the reference (23). The pan-genome analysis was conducted based on the output of Prokka using Roary v 3.12.0 with a BLASTp identity cutoff of 90% (53). The linear map of 10 contigs of 10 *E. anophelis* strains was visualized by the CGView Comparison Tool (54). Amino acid multiple sequence alignments were performed using the ClustalW program in MEGA 11 and visualized in EsPript 3.0 (55, 56).

### Phylogenetic analysis

Twenty-two representative sequences of the 16S rRNA gene of *Elizabethkingia* and *Cloacibacterium normanense* and one hundred and thirty-eight YclQ-like protein sequences encoded by 136 strains were download from the National Center for Biotechnology Information (NCBI) database with coverage ≥90% and identity >30. One hundred and twenty-one YclNOPQ-like sequences were detected in 121 of 136 strains. The sequences of the 16S rRNA gene were aligned using MAFFT v7.520 (57). The amino acid sequences, including the sequences of the NT06 strain, were aligned using MAFFT v7.520 and trimmed using trimAl v1.4 (58). The tandem sequences for YclN, YclO, YclP, and YclQ were obtained using Phylosuite v1.2.2 (59). The maximum likelihood phylogenetic analyses of the 16S rRNA gene, YclQ-like, and YclNOPQ-like sequences were performed using IQ-TREE v2.2.6 with the TPM3 +I + R2, LG + F + I + G4, and LG + F + I + R5 models (1,000 ultrafast bootstraps) respectively, and then illustrated by iTOL v6.6 (60, 61).

## Data Availability

The genome sequences of the 10 *E. anophelis* strains are deposited in the NCBI database under BioProject accession number PRJNA1133835.

## SUPPLEMENTAL MATERIAL

Figure S1 spectrum.01780-24-s0001.pdf The heatmap and cluster result of ANI values among *E. anophelis* strains.

Figure S2 spectrum.01780-24-s0002.pdf Pan-genome characterization based on the distribution of functional categories.

Figure S3 spectrum.01780-24-s0003.pdf The linear map of contig4 of NT06 compared with whole sequences of other 9 *E. anophelis* strains.

Figure S4 spectrum.01780-24-s0004.pdf Concatenated protein tree.

Figure S5 spectrum.01780-24-s0005.pdf 16S rRNA gene phylogenetic tree.

Supplemental tables spectrum.01780-24-s0006.docx Tables S1 to S4.

Table S5 spectrum.01780-24-s0007.xlsx The YclQ-like proteins download from the NCBI database.

ASM does not own the copyrights to Supplemental Material that may be linked to, or accessed through, an article. The authors have granted ASM a non-exclusive, world-wide license to publish the Supplemental Material files. Please contact the corresponding author directly for reuse.

## References

[1] Kim KK, Kim MK, Lim JH, Park HY, Lee ST. 2005. Transfer of Chryseobacterium meningosepticum and Chryseobacterium miricola to Elizabethkingia gen. nov. as Elizabethkingia meningoseptica comb. nov. and Elizabethkingia miricola comb. nov. Int J Syst Evol Microbiol 55:1287–1293. doi:10.1099/ijs.0.63541-0 15879269

[2] Kämpfer P, Matthews H, Glaeser SP, Martin K, Lodders N, Faye I. 2011. Elizabethkingia anophelis sp. nov., isolated from the midgut of the mosquito Anopheles gambiae. Int J Syst Evol Microbiol 61:2670–2675. doi:10.1099/ijs.0.026393-0 21169462

[3] Nascimento APA, de Farias BO, Gonçalves-Brito AS, Magaldi M, Flores C, Quidorne CS, Montenegro KS, Bianco K, Clementino MM. 2023. Phylogenomics analysis of multidrug-resistant Elizabethkingia anophelis in industrial wastewater treatment plant. J Appl Microbiol 134:lxad215. doi:10.1093/jambio/lxad215 37715335

[4] Andriyanov PA, Zhurilov PA, Kashina DD, Tutrina AI, Liskova EA, Razheva IV, Kolbasov DV, Ermolaeva SA. 2022. Antimicrobial resistance and comparative genomic analysis of Elizabethkingia anophelis subsp. endophytica isolated from raw milk. Antibiotics (Basel) 11:648. doi:10.3390/antibiotics11050648 35625292 PMC9137776

[5] Coyle AL. 2017. Elizabethkingia anophelis: exploring the outbreak of disease in the Midwest. Nursing (Auckl) 47:61–63. doi:10.1097/01.NURSE.0000512887.67622.84 28225402

[6] Guerpillon B, Fangous MS, Le Breton E, Artus M, le Gall F, Khatchatourian L, Talarmin JP, Plesiat P, Jeannot K, Saidani N, Rolland-Jacob G. 2022. Elizabethkingia anophelis outbreak in France. Infect Dis Now 52:299–303. doi:10.1016/j.idnow.2022.05.005 35643388

[7] Commans F, Hayer J, Do BN, Tran TTT, Le TTH, Bui TT, Le HS, Bañuls AL, Bui TS, Nguyen QH. 2024. Whole-genome sequence and resistance determinants of four Elizabethkingia anophelis clinical isolates collected in Hanoi, Vietnam. Sci Rep 14:7241. doi:10.1038/s41598-024-57564-3 38538725 PMC10973501

[8] McTaggart LR, Stapleton PJ, Eshaghi A, Soares D, Brisse S, Patel SN, Kus JV. 2019. Application of whole genome sequencing to query a potential outbreak of Elizabethkingia anophelis in Ontario, Canada. Access Microbiol 1:e000017. doi:10.1099/acmi.0.000017 32974512 PMC7470347

[9] Lee YL, Liu KM, Chang HL, Liao YC, Lin JS, Kung FY, Ho CM, Lin KH, Chen YT. 2022. The evolutionary trend and genomic features of an emerging lineage of Elizabethkingia anophelis strains in Taiwan. Microbiol Spectr 10:e0168221. doi:10.1128/spectrum.01682-21 35044198 PMC8768576

[10] Wang B, Cheng R, Feng Y, Guo Y, Kan Q, Qian A, Zhao L. 2022. Elizabethkingia anophelis: an important emerging cause of neonatal sepsis and meningitis in China. Pediatr Infect Dis J 41:e228–e232. doi:10.1097/INF.0000000000003464 35067644

[11] Honavar AG, David A, Amladi A, Thomas L. 2021. Multidrug-resistant Elizabethkingia anophelis septicemia, meningitis, ventriculitis, and hydrocephalus in a preterm neonate: a rare complication of an emerging pathogen. J Pediatr Neurosci 16:79–81. doi:10.4103/jpn.JPN_45_20 34316316 PMC8276963

[12] Ichiki K, Ooka T, Shinkawa T, Inoue S, Hayashida M, Nakamura D, Akimoto M, Yoshimitsu M, Kawamura H, Nakamura M, Obama Y, Gotoh Y, Hayashi T, Nishi J, Ishitsuka K. 2023. Genomic and phylogenetic characterization of Elizabethkingia anophelis strains: the first two cases of life-threatening infection in Japan. J Infect Chemother 29:376–383. doi:10.1016/j.jiac.2023.01.005 36682607

[13] Bulagonda EP, Manivannan B, Mahalingam N, Lama M, Chanakya PP, Khamari B, Jadhao S, Vasudevan M, Nagaraja V. 2018. Comparative genomic analysis of a naturally competent Elizabethkingia anophelis isolated from an eye infection. Sci Rep 8:8447. doi:10.1038/s41598-018-26874-8 29855598 PMC5981450

[14] Chen PJ, Tan MC, Huang WC, Hsu SY, Chen TL, Yang CY, Kuo SC. 2024. The individual contributions of bla_B_, bla_GOB_ and bla_CME_ on MICs of β-lactams in Elizabethkingia anophelis. J Antimicrob Chemother 79:1577–1580. doi:10.1093/jac/dkae137 38742706 PMC11215548

[15] Chen S, Johnson BK, Yu T, Nelson BN, Walker ED. 2020. Elizabethkingia anophelis: physiologic and transcriptomic responses to iron stress. Front Microbiol 11:804. doi:10.3389/fmicb.2020.00804 32457715 PMC7221216

[16] Zhang C. 2014. Essential functions of iron-requiring proteins in DNA replication, repair and cell cycle control. Protein Cell 5:750–760. doi:10.1007/s13238-014-0083-7 25000876 PMC4180463

[17] Frawley ER, Fang FC. 2014. The ins and outs of bacterial iron metabolism. Mol Microbiol 93:609–616. doi:10.1111/mmi.12709 25040830 PMC4135372

[18] Li Y, Liu Y, Chew SC, Tay M, Salido MMS, Teo J, Lauro FM, Givskov M, Yang L. 2015. Complete genome sequence and transcriptomic analysis of the novel pathogen Elizabethkingia anophelis in response to oxidative stress. Genome Biol Evol 7:1676–1685. doi:10.1093/gbe/evv101 26019164 PMC4494045

[19] Perrin A, Larsonneur E, Nicholson AC, Edwards DJ, Gundlach KM, Whitney AM, Gulvik CA, Bell ME, Rendueles O, Cury J, et al.. 2017. Evolutionary dynamics and genomic features of the Elizabethkingia anophelis 2015 to 2016 Wisconsin outbreak strain. Nat Commun 8:15483. doi:10.1038/ncomms15483 28537263 PMC5458099

[20] Lin JN, Lai CH, Yang CH, Huang YH, Lin HH. 2018. Clinical manifestations, molecular characteristics, antimicrobial susceptibility patterns and contributions of target gene mutation to fluoroquinolone resistance in Elizabethkingia anophelis. J Antimicrob Chemother 73:2497–2502. doi:10.1093/jac/dky197 29846598

[21] Deurenberg RH, Bathoorn E, Chlebowicz MA, Couto N, Ferdous M, García-Cobos S, Kooistra-Smid AMD, Raangs EC, Rosema S, Veloo ACM, Zhou K, Friedrich AW, Rossen JWA. 2017. Application of next generation sequencing in clinical microbiology and infection prevention. J Biotechnol 243:16–24. doi:10.1016/j.jbiotec.2016.12.022 28042011

[22] Chen S, Pham S, Terrapon N, Blom J, Walker ED. 2024. Elizabethkingia anophelis MSU001 isolated from Anopheles stephensi: molecular characterization and comparative genome analysis. Microorganisms 12:1079. doi:10.3390/microorganisms12061079 38930461 PMC11206156

[23] Jain C, Rodriguez-R LM, Phillippy AM, Konstantinidis KT, Aluru S. 2018. High throughput ANI analysis of 90K prokaryotic genomes reveals clear species boundaries. Nat Commun 9:5114. doi:10.1038/s41467-018-07641-9 30504855 PMC6269478

[24] Xu J, Pei D, Nicholson A, Lan Y, Xia Q. 2019. In silico identification of three types of integrative and conjugative elements in Elizabethkingia anophelis strains isolated from around the world. mSphere 4:e00040-19. doi:10.1128/mSphere.00040-19 30944210 PMC6449604

[25] Michaelis C, Berger TMI, Kuhlmann K, Ghulam R, Petrowitsch L, Besora Vecino M, Gesslbauer B, Pavkov-Keller T, Keller W, Grohmann E. 2024. Effect of TraN key residues involved in DNA binding on pIP501 transfer rates in Enterococcus faecalis. Front Mol Biosci 11:1268647. doi:10.3389/fmolb.2024.1268647 38380428 PMC10877727

[26] Bonheyo GT, Hund BD, Shoemaker NB, Salyers AA. 2001. Transfer region of a Bacteroides conjugative transposon contains regulatory as well as structural genes. Plasmid 46:202–209. doi:10.1006/plas.2001.1545 11735369

[27] Xu L, Peng B, He Y, Cui Y, Hu Q, Wu Y, Chen H, Zhou X, Chen L, Jiang M, Zuo L, Chen Q, Wu S, Liu Y, Qin Y, Shi X. 2021. Isolation of Elizabethkingia anophelis from COVID-19 swab kits. Front Microbiol 12:799150. doi:10.3389/fmicb.2021.799150 35058914 PMC8763855

[28] Yasmin M, Rojas LJ, Marshall SH, Hujer AM, Cmolik A, Marshall E, Boucher HW, Vila AJ, Soldevila M, Diene SM, Rolain JM, Bonomo RA. 2023. Characterization of a novel pathogen in immunocompromised patients: Elizabethkingia anophelis-exploring the scope of resistance to contemporary antimicrobial agents and β-lactamase inhibitors. Open Forum Infect Dis 10:fad014. doi:10.1093/ofid/ofad014 PMC9938519 36820316

[29] Amladi A, Lal Y B, Jacob JJ, Anandan S, Veeraraghavan B. 2020. Draft genome sequence of carbapenem-resistant Elizabethkingia anophelis strain BP8467 clinical isolate from India. J Glob Antimicrob Resist 21:200–202. doi:10.1016/j.jgar.2020.04.003 32330579

[30] Teo J, Tan SY-Y, Liu Y, Tay M, Ding Y, Li Y, Kjelleberg S, Givskov M, Lin RTP, Yang L. 2014. Comparative genomic analysis of malaria mosquito vector-associated novel pathogen Elizabethkingia anophelis. Genome Biol Evol 6:1158–1165. doi:10.1093/gbe/evu094 24803570 PMC4041001

[31] Murdock A, Bashar S, White D, Uyaguari-Diaz M, Farenhorst A, Kumar A. 2024. Bacterial diversity and resistome analysis of drinking water stored in cisterns from two First Nations communities in Manitoba, Canada. Microbiol Spectr 12:e0314123. doi:10.1128/spectrum.03141-23 38305192 PMC10913478

[32] Pereira AP, Antunes P, Bierge P, Willems RJL, Corander J, Coque TM, Pich OQ, Peixe L, Freitas AR, Novais C, from the ESCMID Study Group on Food- and Water-borne Infections (EFWISG). 2023. Unraveling Enterococcus susceptibility to quaternary ammonium compounds: genes, phenotypes, and the impact of environmental conditions. Microbiol Spectr 11:e0232423. doi:10.1128/spectrum.02324-23 37737589 PMC10581157

[33] Hu S, Lv Y, Xu H, Zheng B, Xiao Y. 2022. Biofilm formation and antibiotic sensitivity in Elizabethkingia anophelis. Front Cell Infect Microbiol 12:953780. doi:10.3389/fcimb.2022.953780 35967866 PMC9366890

[34] Breurec S, Criscuolo A, Diancourt L, Rendueles O, Vandenbogaert M, Passet V, Caro V, Rocha EPC, Touchon M, Brisse S. 2016. Genomic epidemiology and global diversity of the emerging bacterial pathogen Elizabethkingia anophelis. Sci Rep 6:30379. doi:10.1038/srep30379 27461509 PMC4961963

[35] Herbert A, Hancock CN, Cox B, Schnabel G, Moreno D, Carvalho R, Jones J, Paret M, Geng X, Wang H. 2022. Oxytetracycline and streptomycin resistance genes in Xanthomonas arboricola pv. pruni, the causal agent of bacterial spot in peach. Front Microbiol 13:821808. doi:10.3389/fmicb.2022.821808 35283838 PMC8914263

[36] Huszczynski SM, Hao Y, Lam JS, Khursigara CM. 2020. Identification of the Pseudomonas aeruginosa O17 and O15 O-specific antigen biosynthesis loci reveals an ABC transporter-dependent synthesis pathway and mechanisms of genetic diversity. J Bacteriol 202:e00347-20. doi:10.1128/JB.00347-20 32690555 PMC7484189

[37] Cendrowski S, MacArthur W, Hanna P. 2004. Bacillus anthracis requires siderophore biosynthesis for growth in macrophages and mouse virulence. Mol Microbiol 51:407–417. doi:10.1046/j.1365-2958.2003.03861.x 14756782

[38] Abergel RJ, Zawadzka AM, Raymond KN. 2008. Petrobactin-mediated iron transport in pathogenic bacteria: coordination chemistry of an unusual 3,4-catecholate/citrate siderophore. J Am Chem Soc 130:2124–2125. doi:10.1021/ja077202g 18220393

[39] Zawadzka AM, Kim Y, Maltseva N, Nichiporuk R, Fan Y, Joachimiak A, Raymond KN. 2009. Characterization of a Bacillus subtilis transporter for petrobactin, an anthrax stealth siderophore. Proc Natl Acad Sci U S A 106:21854–21859. doi:10.1073/pnas.0904793106 19955416 PMC2799803

[40] Wick RR, Judd LM, Gorrie CL, Holt KE. 2017. Unicycler: resolving bacterial genome assemblies from short and long sequencing reads. PLoS Comput Biol 13:e1005595. doi:10.1371/journal.pcbi.1005595 28594827 PMC5481147

[41] Gurevich A, Saveliev V, Vyahhi N, Tesler G. 2013. QUAST: quality assessment tool for genome assemblies. Bioinformatics 29:1072–1075. doi:10.1093/bioinformatics/btt086 23422339 PMC3624806

[42] Chklovski A, Parks DH, Woodcroft BJ, Tyson GW. 2023. CheckM2: a rapid, scalable and accurate tool for assessing microbial genome quality using machine learning. Nat Methods 20:1203–1212. doi:10.1038/s41592-023-01940-w 37500759

[43] Seemann T. 2014. Prokka: rapid prokaryotic genome annotation. Bioinformatics 30:2068–2069. doi:10.1093/bioinformatics/btu153 24642063

[44] Bateman A, Martin M-J, Orchard S, Magrane M, Ahmad S, Alpi E, Bowler-Barnett EH, Britto R, Bye-A-Jee H, Cukura A, et al.. 2023. UniProt: the universal protein knowledgebase in 2023. Nucleic Acids Res 51:D523–D531. doi:10.1093/nar/gkac1052 36408920 PMC9825514

[45] Lagesen K, Hallin P, Rødland EA, Staerfeldt HH, Rognes T, Ussery DW. 2007. RNAmmer: consistent and rapid annotation of ribosomal RNA genes. Nucleic Acids Res 35:3100–3108. doi:10.1093/nar/gkm160 17452365 PMC1888812

[46] Lowe TM, Chan PP. 2016. tRNAscan-SE On-line: integrating search and context for analysis of transfer RNA genes. Nucleic Acids Res 44:W54–W57. doi:10.1093/nar/gkw413 27174935 PMC4987944

[47] Aramaki T, Blanc-Mathieu R, Endo H, Ohkubo K, Kanehisa M, Goto S, Ogata H. 2020. KofamKOALA: KEGG Ortholog assignment based on profile HMM and adaptive score threshold. Bioinformatics 36:2251–2252. doi:10.1093/bioinformatics/btz859 31742321 PMC7141845

[48] Huerta-Cepas J, Forslund K, Coelho LP, Szklarczyk D, Jensen LJ, von Mering C, Bork P. 2017. Fast genome-wide functional annotation through orthology assignment by eggNOG-mapper. Mol Biol Evol 34:2115–2122. doi:10.1093/molbev/msx148 28460117 PMC5850834

[49] Alcock BP, Raphenya AR, Lau TTY, Tsang KK, Bouchard M, Edalatmand A, Huynh W, Nguyen A-LV, Cheng AA, Liu S, et al.. 2020. CARD 2020: antibiotic resistome surveillance with the comprehensive antibiotic resistance database. Nucleic Acids Res 48:D517–D525. doi:10.1093/nar/gkz935 31665441 PMC7145624

[50] Altschul SF, Gish W, Miller W, Myers EW, Lipman DJ. 1990. Basic local alignment search tool. J Mol Biol 215:403–410. doi:10.1016/S0022-2836(05)80360-2 2231712

[51] Liu B, Zheng D, Zhou S, Chen L, Yang J. 2022. VFDB 2022: a general classification scheme for bacterial virulence factors. Nucleic Acids Res 50:D912–D917. doi:10.1093/nar/gkab1107 34850947 PMC8728188

[52] Bertelli C, Laird MR, Williams KP, Lau BY, Hoad G, Winsor GL, Brinkman FSL, Simon Fraser University Research Computing Group. 2017. IslandViewer 4: expanded prediction of genomic islands for larger-scale datasets. Nucleic Acids Res 45:W30–W35. doi:10.1093/nar/gkx343 28472413 PMC5570257

[53] Page AJ, Cummins CA, Hunt M, Wong VK, Reuter S, Holden MTG, Fookes M, Falush D, Keane JA, Parkhill J. 2015. Roary: rapid large-scale prokaryote pan genome analysis. Bioinformatics 31:3691–3693. doi:10.1093/bioinformatics/btv421 26198102 PMC4817141

[54] Petkau A, Stuart-Edwards M, Stothard P, Van Domselaar G. 2010. Interactive microbial genome visualization with GView. Bioinformatics 26:3125–3126. doi:10.1093/bioinformatics/btq588 20956244 PMC2995121

[55] Tamura K, Stecher G, Kumar S. 2021. MEGA11: molecular evolutionary genetics analysis version 11. Mol Biol Evol 38:3022–3027. doi:10.1093/molbev/msab120 33892491 PMC8233496

[56] Robert X, Gouet P. 2014. Deciphering key features in protein structures with the new ENDscript server. Nucleic Acids Res 42:W320–W324. doi:10.1093/nar/gku316 24753421 PMC4086106

[57] Katoh K, Standley DM. 2013. MAFFT multiple sequence alignment software version 7: improvements in performance and usability. Mol Biol Evol 30:772–780. doi:10.1093/molbev/mst010 23329690 PMC3603318

[58] Capella-Gutiérrez S, Silla-Martínez JM, Gabaldón T. 2009. trimAl: a tool for automated alignment trimming in large-scale phylogenetic analyses. Bioinformatics 25:1972–1973. doi:10.1093/bioinformatics/btp348 19505945 PMC2712344

[59] Zhang D, Gao F, Jakovlić I, Zou H, Zhang J, Li WX, Wang GT. 2020. PhyloSuite: an integrated and scalable desktop platform for streamlined molecular sequence data management and evolutionary phylogenetics studies. Mol Ecol Resour 20:348–355. doi:10.1111/1755-0998.13096 31599058

[60] Nguyen L-T, Schmidt HA, von Haeseler A, Minh BQ. 2015. IQ-TREE: a fast and effective stochastic algorithm for estimating maximum-likelihood phylogenies. Mol Biol Evol 32:268–274. doi:10.1093/molbev/msu300 25371430 PMC4271533

[61] Letunic I, Bork P. 2024. Interactive tree of life (iTOL) v6: recent updates to the phylogenetic tree display and annotation tool. Nucleic Acids Res 52:W78–W82. doi:10.1093/nar/gkae268 38613393 PMC11223838

