# Supplementary figures and images for: Comparative genomic analysis of ten Elizabethkingia anophelis isolated from clinical patients in China
Source: Microbiol Spectr. 2024 Nov 29;13(1):e01780-24. doi: 10.1128/spectrum.01780-24 (PMC11705823; doi:10.1128/spectrum.01780-24)

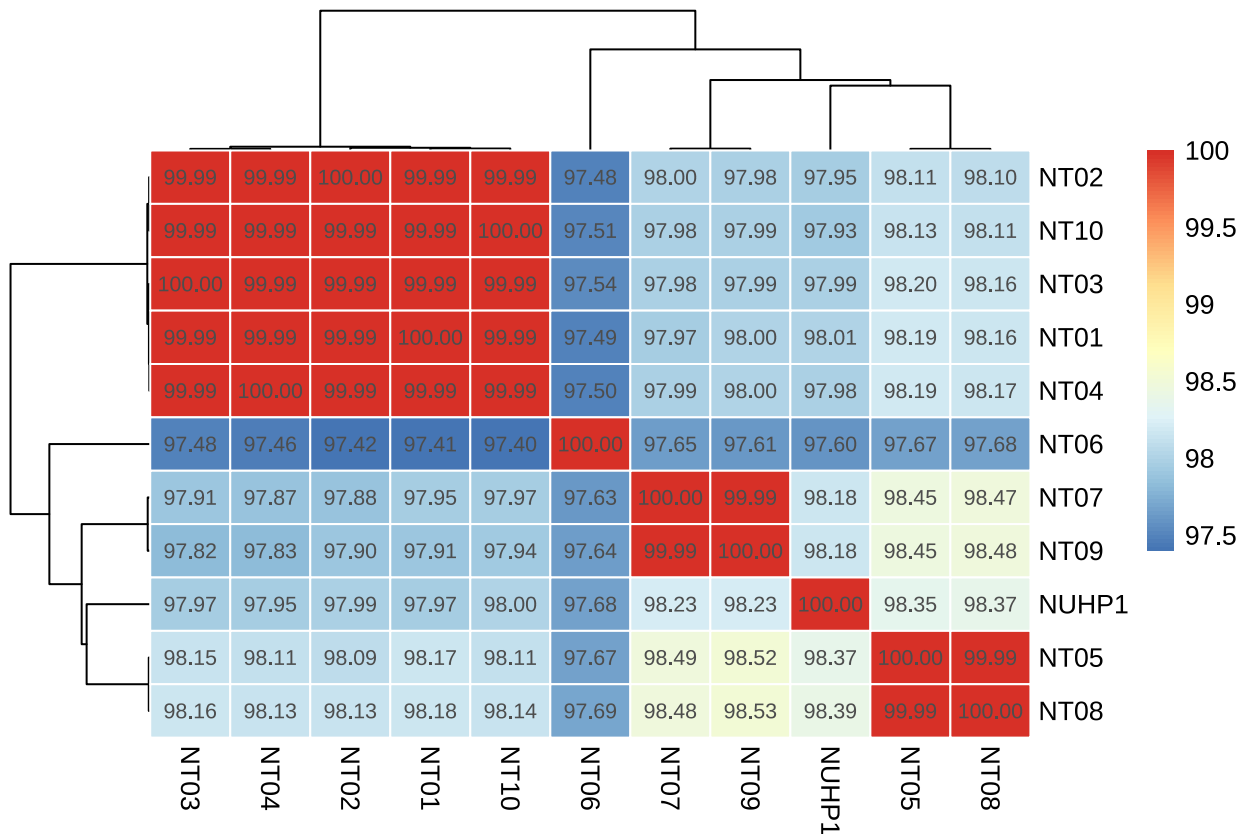

**FIG S1** The heatmap and cluster result of ANI values among *E. anophelis* strains.

Supplement: Figure S1 — The heatmap and cluster result of ANI values among E. anophelis strains. [file spectrum.01780-24-s0001.pdf]
