# Supplementary material for: Comparative genomic analysis of ten Elizabethkingia anophelis isolated from clinical patients in China
Source: Microbiol Spectr. 2024 Nov 29;13(1):e01780-24. doi: 10.1128/spectrum.01780-24 (PMC11705823; doi:10.1128/spectrum.01780-24)
Supplement: Figure S2 — Pan-genome characterization based on the distribution of functional categories. [file spectrum.01780-24-s0002.pdf]

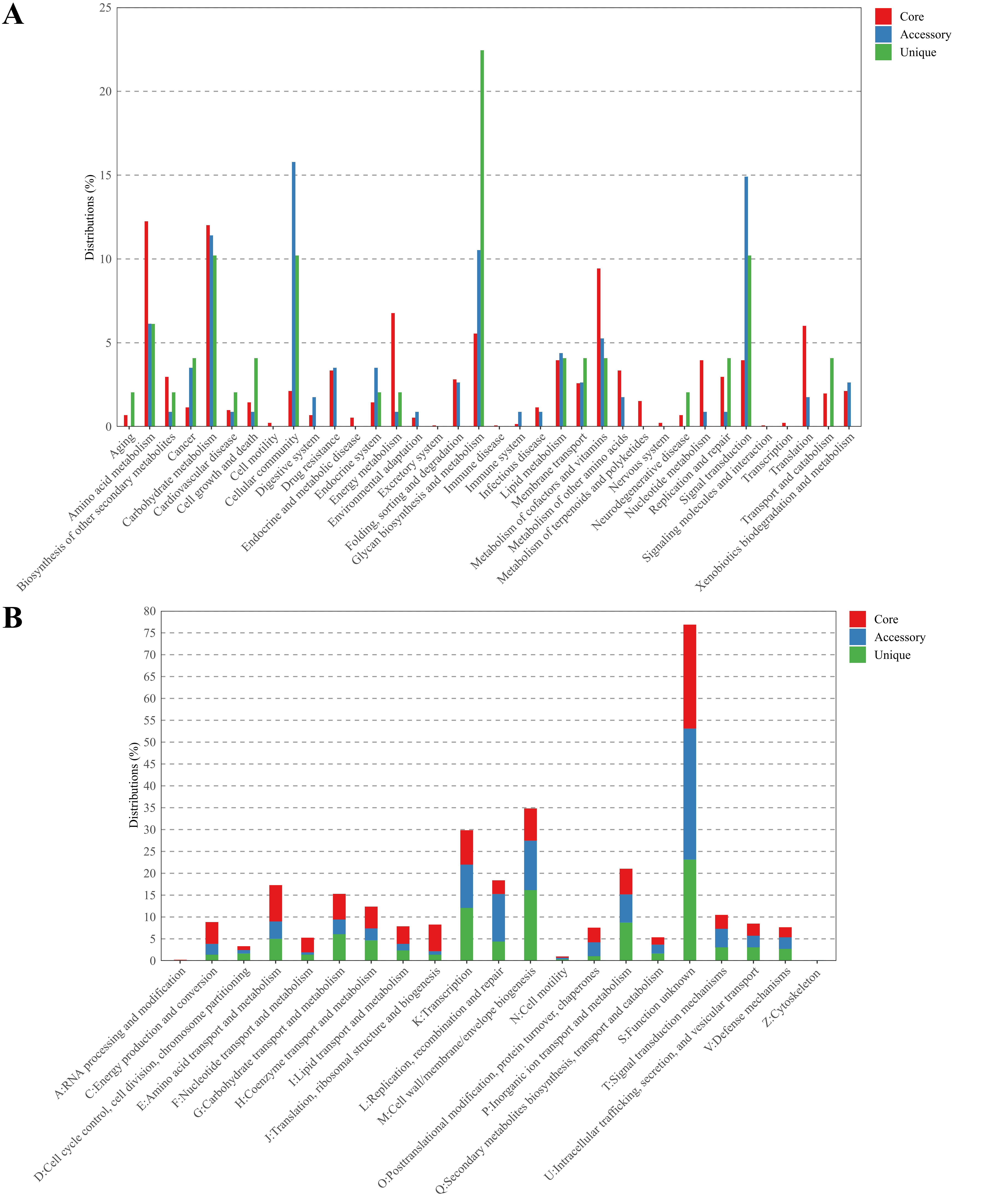

FIG S2 Pan-genome characterization based on the distribution of functional categories. Distribution of KEGG (A) and COG (B) categories in the core, accessory, and unique genomes.
