# Supplementary material for: Comparative genomic analysis of ten Elizabethkingia anophelis isolated from clinical patients in China
Source: Microbiol Spectr. 2024 Nov 29;13(1):e01780-24. doi: 10.1128/spectrum.01780-24 (PMC11705823; doi:10.1128/spectrum.01780-24)
Supplement: Figure S3 — The linear map of contig4 of NT06 compared with whole sequences of other 9 E. anophelis strains. [file spectrum.01780-24-s0003.pdf]

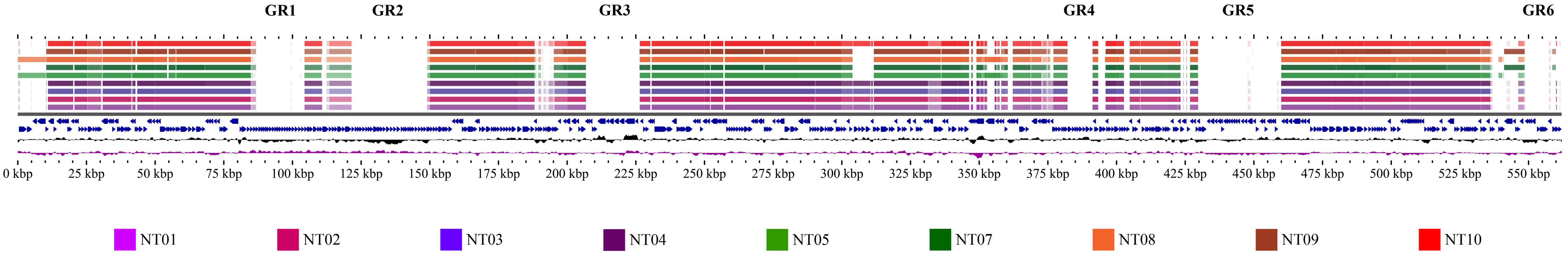


**FIG S3** The linear map of contig4 of NT06 compared with whole sequences of other 9 *E. anophelis* strains. Contig4 was used as reference sequences.
