# Supplementary material for: Comparative genomic analysis of ten Elizabethkingia anophelis isolated from clinical patients in China
Source: Microbiol Spectr. 2024 Nov 29;13(1):e01780-24. doi: 10.1128/spectrum.01780-24 (PMC11705823; doi:10.1128/spectrum.01780-24)
Supplement: Figure S4 — Concatenated protein tree. [file spectrum.01780-24-s0004.pdf]

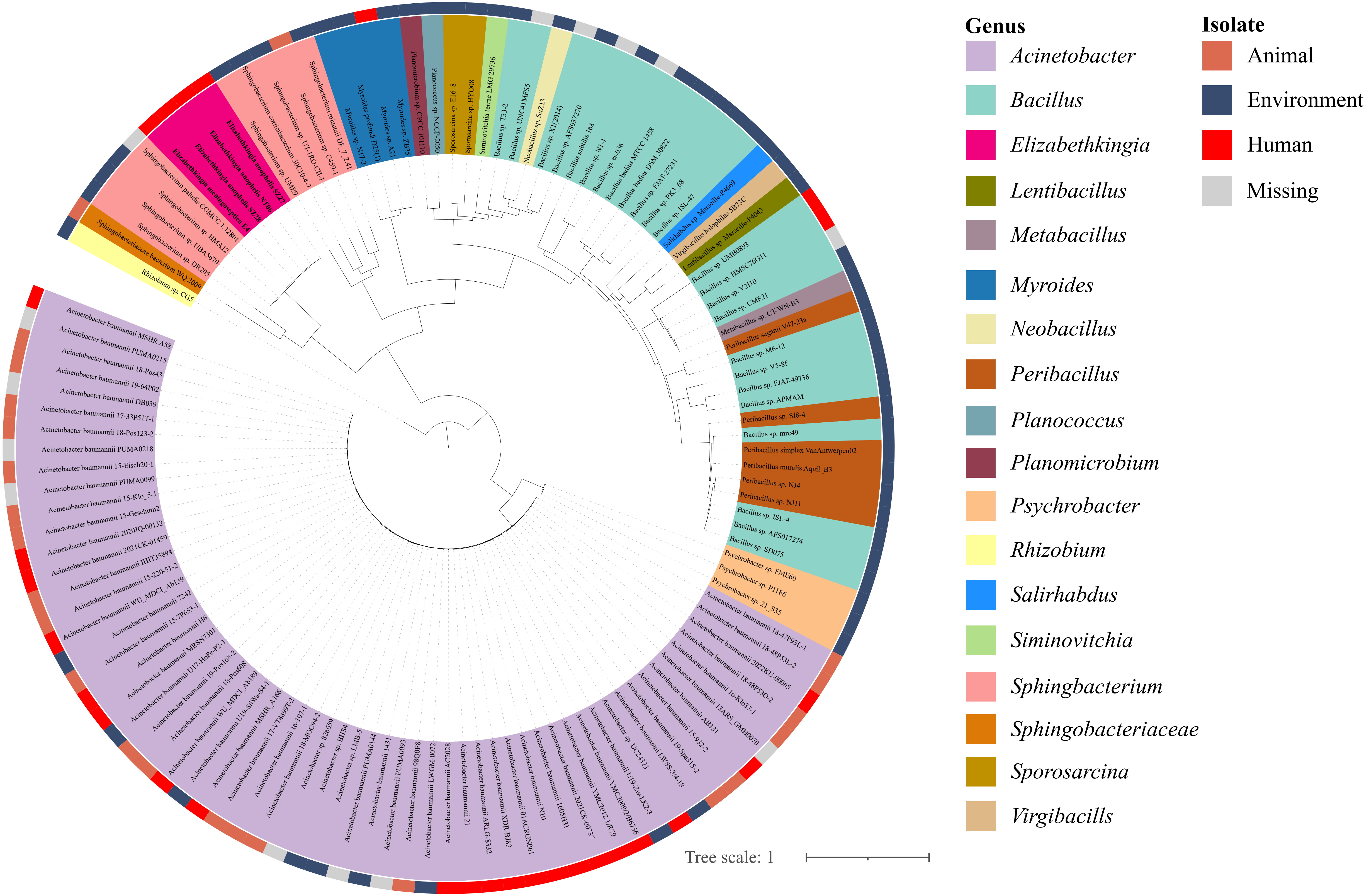

FIG S4 Concatenated protein tree. Maximum likelihood phylogenetic analysis of 122 YclNOPQ-like protein sequences from 122 strains. The protein sequences were aligned using MAFFT, and the alignments were trimmed using trimAl. The tandem sequences were obtained by Phylosuite v1.2.2. The tree was made using IQ-TREE and *R. sp*. CG5 was used as the outgroup. The different colors represent different genera and isolations, as defined in the legend.
