# Supplementary material for: Comparative genomic analysis of ten Elizabethkingia anophelis isolated from clinical patients in China
Source: Microbiol Spectr. 2024 Nov 29;13(1):e01780-24. doi: 10.1128/spectrum.01780-24 (PMC11705823; doi:10.1128/spectrum.01780-24)
Supplement: Figure S5 — 16S rRNA gene phylogenetic tree. [file spectrum.01780-24-s0005.pdf]

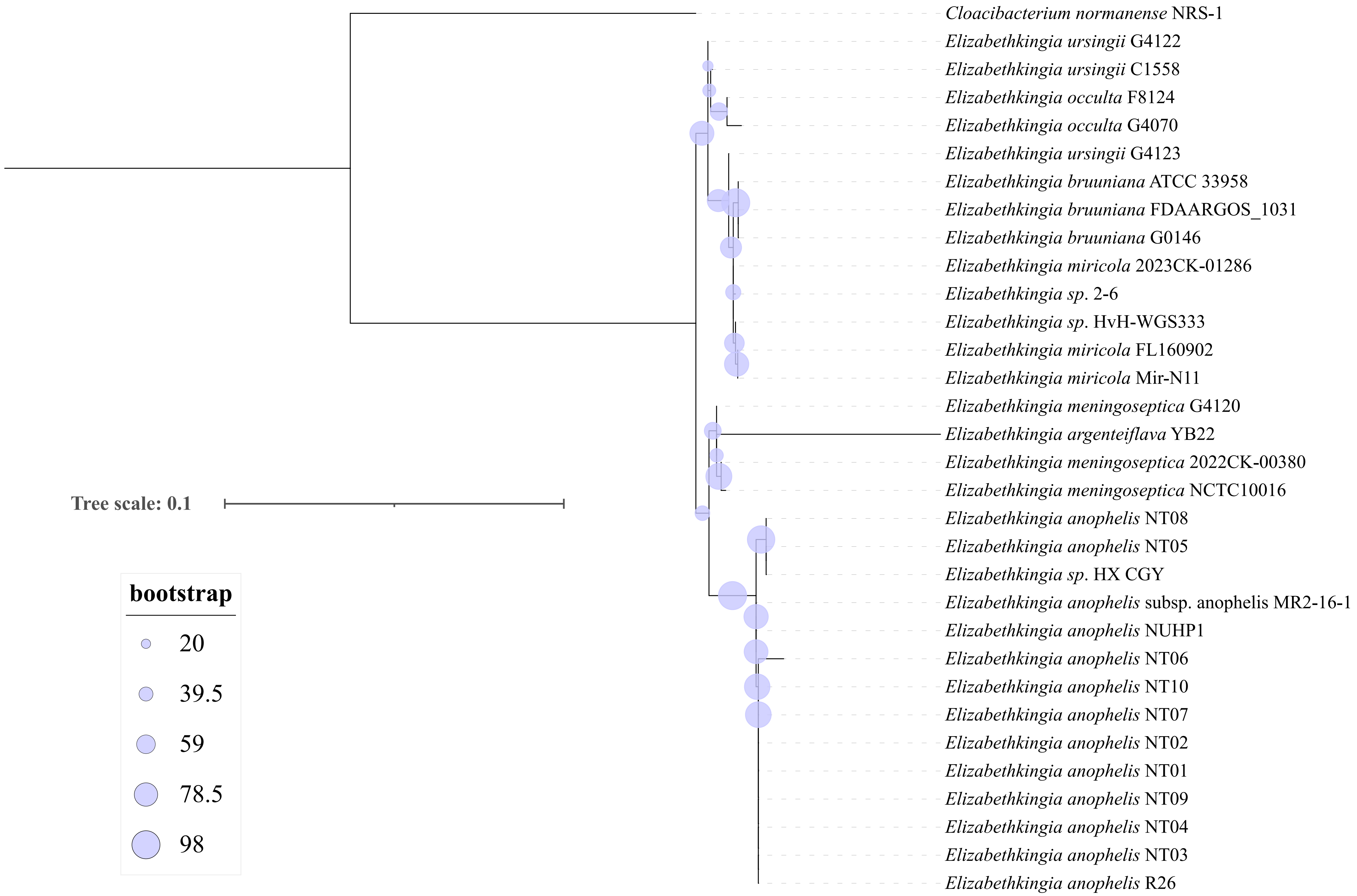

FIG S5 16S rRNA gene phylogenetic tree obtained in IQ-TREE from the 16S rRNA gene sequences of *Elizabethkingia* and their related taxa using maximum likelihood method. *Cloacibacterium normanense* NRS-1was used as outgroup. The circle on the nodes indicates the bootstrap value.
