# Supplementary material for: Comparative genomic analysis of ten Elizabethkingia anophelis isolated from clinical patients in China
Source: Microbiol Spectr. 2024 Nov 29;13(1):e01780-24. doi: 10.1128/spectrum.01780-24 (PMC11705823; doi:10.1128/spectrum.01780-24)
Supplement: Supplemental tables — Tables S1 to S4. [file spectrum.01780-24-s0006.docx]

| **Table S1** The pathway of unique genes of NT06 strain | | |
| --- | --- | --- |
| KEGG pathways | Number |  |
| Aging | 1 |  |
| Amino acid metabolism | 3 |  |
| Biosynthesis of other secondary metabolites | 1 |  |
| Cancer | 2 |  |
| Carbohydrate metabolism | 5 |  |
| Cardiovascular disease | 1 |  |
| Cellular community | 5 |  |
| Endocrine system | 1 |  |
| Energy metabolism | 1 |  |
| Glycan biosynthesis and metabolism | 11 |  |
| Lipid metabolism | 2 |  |
| Membrane transport | 2 |  |
| Metabolism of cofactors and vitamins | 2 |  |
| Neurodegenerative disease | 1 |  |
| Signal transduction | 5 |  |
| Transport and catabolism | 2 |  |

| **Table S2** The COG functions of unique genes of NT06 strain | | |
| --- | --- | --- |
| Classification of COG functions | Number |  |
| N: Cell motility | 1 |  |
| F: Nucleotide transport and metabolism | 2 |  |
| O: Posttranslational modification, protein turnover, chaperones | 3 |  |
| C: Energy production and conversion | 4 |  |
| D: Cell cycle control, cell division, chromosome partitioning | 4 |  |
| J: Translation, ribosomal structure and biogenesis | 4 |  |
| Q: Secondary metabolites biosynthesis, transport and catabolism | 5 |  |
| V: Defense mechanisms | 6 |  |
| I: Lipid transport and metabolism | 7 |  |
| U: Intracellular trafficking, secretion, and vesicular transport | 7 |  |
| T: Signal transduction mechanisms | 9 |  |
| L: Replication, recombination and repair | 10 |  |
| E: Amino acid transport and metabolism | 14 |  |
| H: Coenzyme transport and metabolism | 14 |  |
| G: Carbohydrate transport and metabolism | 17 |  |
| P: Inorganic ion transport and metabolism | 25 |  |
| K: Transcription | 35 |  |
| M: Cell wall/membrane/envelope biogenesis | 46 |  |
| S: Function unknown | 66 |  |

**TABLE S3** Characteristics of six genomic regions (GRs) presented among the NT06 strain

| Genomic region | Start | End | Size (nt) | Features |
| --- | --- | --- | --- | --- |
| GR1 | 86,732 | 104,317 | 17,585 | glycosyltransferase family protein, beta-1,6-N-acetylglucosaminyltransferase,serine acetyltransferase |
| GR2 | 121,509 | 148,981 | 27,472 | O-antigen polysaccharide polymerase Wzy, N-acetyl sugar amidotransferase, imidazole glycerol phosphate synthase subunit HisH,UDP-N-acetylglucosamine 2-epimerase, glycosyltransferase family protein |
| GR3 | 206,843 | 226,340 | 19,497 | hexameric tyrosine-coordinated heme protein, AAA family ATPase |
| GR4 | 382,162 | 391,260 | 9,098 | Na+/H+ antiporter, siderophore-interacting protein,universal stress protein UspA |
| GR5 | 429,707 | 459,917 | 30,210 | conjugal transfer protein MobABC, AraC family transcriptional regulator, Ferric-anguibactin-binding protein FatB, Petrobactin import ATP-binding protein YclP,Petrobactin import system permease protein YclO, Petrobactin import system permease protein YclN |
| GR6 | 548,588 | 558,627 | 10,039 | polysaccharide deacetylase family protein, glycosyltransferase family 2 protein, phosphatase PAP2 family protein |

**TABLE S4** The result of BLASTx of *ycl*-like cluster genes in the NT06 against Ycl proteins *Bacillus subtilis* 168

| Gene id | Protein name | Coverage (%) | Identity (%) |
| --- | --- | --- | --- |
| NT06_02217 | YclQ | 92.60 | 39.00 |
| NT06_02218 | YclP | 99.21 | 51.00 |
| NT06_02219 | YclO | 88.29 | 38.93 |
| NT06_02220 | YclN | 93.90 | 43.51 |
